# Understanding the implementation of evidence-based care: A structural network approach

**DOI:** 10.1186/1748-5908-6-14

**Published:** 2011-02-24

**Authors:** Michael L Parchman, Caterina M Scoglio, Phillip Schumm

**Affiliations:** 1 Family & Community Medicine Department, 7703 Floyd Curl Drive, University of Texas Health Science Center, San Antonio, Texas, 78229-3884, USA; 2 VERDICT Health Services Research Program (11C6), South Texas Veterans Healthcare System, 7400 Merton Minter Blvd, San Antonio, TX 78229-4404, USA; 3 Electrical and Computer Engineering Department, 2069 Rathbone Hall, Kansas State University, Manhatten, KS 66506, USA

## Abstract

**Background:**

Recent study of complex networks has yielded many new insights into phenomenon such as social networks, the internet, and sexually transmitted infections. The purpose of this analysis is to examine the properties of a network created by the 'co-care' of patients within one region of the Veterans Health Affairs.

**Methods:**

Data were obtained for all outpatient visits from 1 October 2006 to 30 September 2008 within one large Veterans Integrated Service Network. Types of physician within each clinic were nodes connected by shared patients, with a weighted link representing the number of shared patients between each connected pair. Network metrics calculated included edge weights, node degree, node strength, node coreness, and node betweenness. Log-log plots were used to examine the distribution of these metrics. Sizes of k-core networks were also computed under multiple conditions of node removal.

**Results:**

There were 4,310,465 encounters by 266,710 shared patients between 722 provider types (nodes) across 41 stations or clinics resulting in 34,390 edges. The number of other nodes to which primary care provider nodes have a connection (172.7) is 42% greater than that of general surgeons and two and one-half times as high as cardiology. The log-log plot of the edge weight distribution appears to be linear in nature, revealing a 'scale-free' characteristic of the network, while the distributions of node degree and node strength are less so. The analysis of the k-core network sizes under increasing removal of primary care nodes shows that about 10 most connected primary care nodes play a critical role in keeping the *k*-core networks connected, because their removal disintegrates the highest *k*-core network.

**Conclusions:**

Delivery of healthcare in a large healthcare system such as that of the US Department of Veterans Affairs (VA) can be represented as a complex network. This network consists of highly connected provider nodes that serve as 'hubs' within the network, and demonstrates some 'scale-free' properties. By using currently available tools to explore its topology, we can explore how the underlying connectivity of such a system affects the behavior of providers, and perhaps leverage that understanding to improve quality and outcomes of care.

## Background

Efforts to date to understand the slowness of physicians to implement evidence-based guidelines has been hindered by an overreliance on the attributes, knowledge, decision making, and actions of individual clinicians and an under-recognition of the network of care within which they operate [1-5]. For example, in efforts to understand adoption of guidelines, research to date has largely focused on individual attributes of the providers using theories such as the theory of planned behavior [6]. However, little is known about adoption of guidelines from the perspective of the network of providers within which a single provider is embedded.

One of the earliest examinations of diffusion of information and behaviors between physicians is the landmark study of physician prescribing behavior by Coleman, Katz and Mentzel in the mid-1950s [7]. They found that the properties of relationships formed by physicians in a network predict the adoption of a new medication. The adoption occurs first between community physicians who have contact with opinion leaders, and then between physicians who are social friends. However, re-analysis of the data raised questions about the findings and how the opinions and behaviors of other physicians affect those with whom they interact [8].

Physicians may also influence each other as they observe and compare the care provided to their patients by other physicians, even if they have no direct communication with the other physician. As noted by Mittman and colleagues, healthcare professionals work within peer groups who share common values, assumptions, and beliefs, and individual behavior can be strongly influenced by these factors [3]. Patients often return to their physician after contact with another physician with a new diagnostic workup, or taking a new medication the primary physician may not be familiar or comfortable with. For example, Keating and colleagues documented that physicians obtain information from other physicians who they consider to have more expertise in the knowledge area [9].

The 'sharing of care' between two physicians creates a link or a connection. Physicians who share the care of many patients have stronger linkages than with physicians whom they share the care of few patients. Physicians are also connected with many other physicians through these linkages, all of which when taken into consideration form a 'network of healthcare delivery.' What is not well understood is if this pattern of shared care influences the awareness, acceptance, and adoption of new information by physicians across an integrated network. To examine this issue, it is necessary to establish the feasibility of constructing such a network and examine its properties before testing hypotheses about how these network metrics or properties might influence provider behavior and healthcare outcomes.

**Figure 1:**
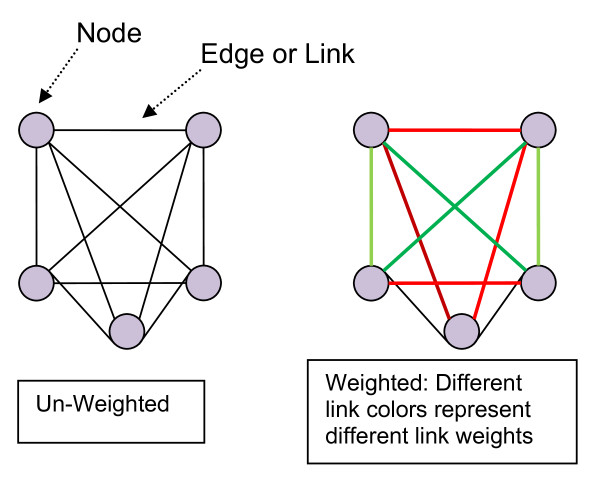
**Undirected network diagrams**.

Over the past 15 years, there has been an explosion of interest in the study of complex networks [10]. Network science has advanced our understanding of complex systems from the internet and worldwide web, social systems, and organizations [11], all the way down to the protein communication channels within cells [12]. Most network research is an outgrowth of graph theory, a field within discrete mathematics [13,14]. A defined set of entities, designated as 'nodes,' are represented as vertices on the graph. Relationships between the nodes are represented as links or 'edges' (Figure 1). This representational framework, although on its surface quite basic, can be remarkably complex. For example, edges can be non-directional, unidirectional, or bidirectional. Edges can have weights which represent some strength of the relationship between two nodes. By calculating how many edges connect a node to the network, the strength of the connection of a node to the network can be determined. In the network created in this paper, the strengths of the edges are calculated as the number of patients two physicians have in common. Although node degree and edge weight can tell us about how well a node is connected to the network, nodes can have relative positions within the network represented by measures of centrality. For example, the '*k*-coreness' of a node is defined as the presence of the node in a sub-network obtained by stepwise removal of nodes that are less well connected to the network as measured by their node degree for unweighted networks or strength for weighted ones. The *k*-core sub-networks are comprised of nodes with a remaining node degree or strength of *k *or higher.

These network metrics or properties also reflect the rules governing network formation. The first well-studied network models, namely Erdos-Renyi and Gilbert random graphs, assumed that the connections between nodes in a network were generated randomly, with a given probability. More recent work has established new network models that are formed by 'preferential attachment' of new nodes [15]. Preferential attachment describes a phenomenon where the probability that a node is connected to another node is proportional to the other node's degree, strength, or other measure of connectivity, or more generally the node's wealth. This follows the 'rich get richer' cliché. One result of a network whose formation is governed by preferential attachment is that the distribution of network metrics or properties follows a power-law. Interestingly, it has been shown that many real world complex networks are well represented by preferential attachment models [15].

There have been some attempts to examine the delivery of healthcare from a network perspective [9,16,17]. Iwashyna and colleagues describe a critical care network comprised of hospitals [17]. Others have described a relational approach to competition between hospitals [16], and a social network of physicians within one academic health center based on who they say they go to for advice about women's health issues [9]. However, creating a network comprised of clinicians who are connected to each other by the shared care of a patient has, to our knowledge, not been used to study the complex network of healthcare delivery. The purpose of this analysis is to examine the properties of a complex network formed by the delivery of outpatient care within one regional Veterans Healthcare System, a 'Veterans Health Administration Veterans Integrated Service Network' (VISN) and explore the implications of these properties for implementation of new evidence into medical practice.

## Sources of data

The Department of Veterans Affairs (VA) is the largest integrated healthcare delivery system in the US. Because it has an integrated electronic health system used by all clinicians, it is an ideal setting to examine the network properties of outpatient healthcare delivery. The VA divides its national delivery system into regional systems called VISNs. Each VISN has two or more VA medical centers with outlying outpatient clinics. Within each clinic, physicians may refer to each other or to physicians at another clinic or VA medical center within the VISN. The VA system has a clear hierarchical structure, which will be reflected in the network's structure.

For purposes of this study, we obtained VA administrative data on all outpatient encounters from one VISN with three VA medical centers over a 36-month time period: 1 October 2006 to 30 September 2008. This data set provides 'station' or clinic location of service, 'provider type' within each station, and a patient identifier and date of service along with the diagnoses for each service delivered. For data security purposes, identifiers of the clinic and individual patients were scrambled so they were de-identified. Date of service for each patient was randomly offset to prevent identification as well. In addition, the VA would not allow identification of individual healthcare providers, only the type of provider within each clinic or 'station' in this VISN. Therefore, one node in the network may represent one cardiologist or many cardiologists within the same clinic location.

## Network construction

The network was constructed based on the following rules: Each node is a physician type within a clinic location; an edge between two nodes represents one or more patients who have visited both clinician types and is weighted by the number of shared patients. There are many possible networks that could be constructed using the available data. Because the objective of this analysis is to investigate the spread of information, this 'co-care' network was selected based on the work previously mentioned demonstrating that physicians within a healthcare setting share information with each other about patient care and influence each other's opinions [3,9]. The relationships among provider nodes can be estimated by the sharing of patients.

## Network measurements

This section summarizes the different mathematical approaches to measure the topological characteristics of the network of the VA outpatient care. Many diverse metrics have been proposed in the literature by authors from multiple disciplines such as discrete mathematics, statistical physics, and networking to assess *a priori *strengths and weaknesses of networks [13,14]. However, the specificity of information provided by each metric is not clear, because the information is partial and interdependent. For the question of diffusion, one might examine degree centrality, betweenness centrality, or a key actor formulation in an effort to understand how information and changes in provider behavior are influenced by the network in which they are embedded [18,19]. In the following, we describe four network metrics selected for the purposes of this analysis.

## Node degree

The degree *d_v _*of a given node *v *is defined as the number of links connected to it. The degree also is equal to the number of nodes that are at distance one from *v*, also called neighbors of *v*. Computing *d_v _*for each *v*, we can deduce the node degree distribution. Typical node degree distributions for large real world networks show a heavy tail. This means that there are a few, but not zero, nodes with very high node degrees. These nodes are frequently called hubs, and play a critical role in the network.

## Node strength and edge weights

Edges themselves can also have weights, which are considered to be the strength of the link between nodes. So each link between a pair of physicians can be thought of as having a weight determined by the number of unique patients shared between these two physicians over a given period of time, and this value can be determined for each edge, or across a set of edges within a sub-network. Using edge weights, the concept of node degree is extended to define the strength of the node as the total weights of the links connected to it. All metrics can be extended to the case of weighted networks, and a thorough definition and discussion of them can be found in Barrat *et al*. and Newman [13,14].

## Node betweenness

Between every pair of nodes in a connected network, there exists a path on the network among all possible paths that has the shortest distance between the pair of nodes. For each node, the number of shortest paths that transverse a node, normalized by the maximum possible number of shortest paths that could traverse the node, is known as node betweenness, and serves as a centrality measure [13,14]. To compute distance-based paths in this network, the inverse of the edge weight is used to represent the 'distance' between one connected pair of nodes: a higher number of shared patients between two provider nodes corresponds to a shorter distance between them.

## Node 'coreness'

This definition of the coreness or centrality of a node within a larger network is based on the decomposition of the network in its *k*-core sub-networks. This decomposition is obtained by pruning iteratively the least connected nodes, thus detecting the nodes that progressively belong to the central core. The *k*-core sub-network of a network can be obtained by recursively removing all nodes of degree less than *k*, until all nodes in the remaining network have at least degree *k*. After the iterative removal of all nodes of degree less than *k*, the size of the remaining *k*-core sub-network is the number of nodes remaining, where *k *is referred to as the threshold of the *k*-core. A node is said to have coreness *k *if it belongs to the *k*-core but does not belong to the (*k*+1)-core [13,14].

## Analysis

The network was constructed by linking provider types within and between each station/clinic (nodes) together with links (edges) that are shared patients. The data set was limited to provider types that only represent clinicians (physicians, physician assistants, and nurse practitioners) and excluded encounters such as nurse phone calls or pharmacy medication pick-ups. Furthermore, we eliminated resident or fellows from the analysis because of their rapid turnover from one year to the next. The network metrics were calculated using C. Visualization of the resulting network was enabled through the KiNG software. http://kinemage.biochem.duke.edu The distribution of each of the network properties was plotted because the resulting plots inform us about both how the network was formed and the topology of the network itself [13].

## Results

The initial data set included all outpatient encounters, including phone calls, pharmacy pick-up, and nurse entries into the medical record. Using provider type codes we were able to limit the data set to encounters with physicians, physician assistants, and nurse practitioners. This reduced data set had 4,310,465 encounters by 266,710 shared patients between 722 provider nodes across 41 stations or clinics resulting in 34,390 edges. It is important to remember that a link between any two provider nodes can occur with one shared patient or many shared patients. Thus these links or 'edges' have weights that correspond to the strength of the connection between any two nodes which represent a provider type within a clinic/station.

**Figure 2:**
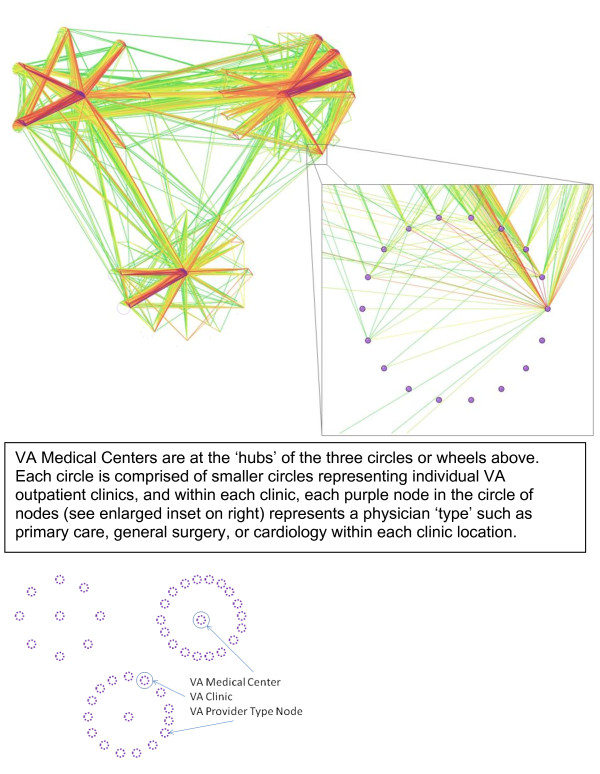
**Network diagram of all physician types across clinics who shared a patient over a 36 month time period, showing only connections composed of more than 10 patients**.

The graphical description of the resulting network is shown in Figure 2. This network is organized around the three VA medical centers within the VISN. Each 'node' in the figure represents a provider or clinician type within a clinic location, *e.g*., primary care, general surgery, or cardiology. The only edges or links displayed in Figure 2 are those where more than 10 patients are shared between any two provider nodes within a station. As the colors of the edges move from greens to reds to violets, the edges represent higher numbers of shared patients between two nodes, and thus higher 'edge weights.' We grouped these provider node types by location such that each circle of nodes represents a clinic or station (See the inset enlarged clinic in Figure 2). These circles of nodes representing clinics or stations are then arranged into three larger circles of clinics by their association with the three VA medical center clinics, which are located in the centers of each of the three large circles. The orientation of the network in Figure 2 displays a view of the organizational structure of the VISN.

Results of the calculation of network metrics are shown in Table 1. These results suggest that primary care nodes are very well connected 'central' nodes in the network. In fact, some of them with very high node degrees function as 'hubs,' which are very highly connected nodes in the network. In fact, their node degree, the number of other nodes or provider types to which they have a connection by sharing a patient (172.7), is 42% greater than that of general surgeon nodes and two and one-half times as high as cardiology nodes. Similar magnitudes of difference are found with node strength and edge weight. Of the top 20 nodes as ranked by node strength, 10 were primary care, five were surgeon nodes and three were cardiology nodes and two were rehabilitation nodes. As mentioned in the methods, one might also examine betweeness centrality rather than degree centrality. We calculated both to compare results and found similar results with the primary care nodes having an average value three times greater than that of general surgeon nodes, who had the second highest average. Both degree centrality and betweenness centrality identify the primary care provider nodes as the most central set of nodes.

**Table 1:** Network metrics

	Mean (S.D)	Median	Range
Node Degree			
All Providers	95.3 (93.3)	72.5	1 to 429
Primary Care	172.7 (100.5)	163.5	1 to 429
General Surgery	121.4 (114.0)	108	1 to 400
Cardiology	66.9 (117.1)	14	1 to 353
Pulmonary	75.6 (106.3)	12.5	1 to 318

Node Strength			
All Providers	3,885.1 (9,345.9)	432	1 to 103,618
Primary Care	11,121.4 (18,481.8)	4,410.5	1 to 103,618
General Surgery	6,560.1 (11,273.2)	1,175.5	1 to 59,696
Cardiology	5,314.9 (13,090.8)	28	1 to 47,045
Pulmonary	3,726.6 (8,559.4)	15	1 to 33,070

Edge Weight			
All Edges	40.8 (199.6)	3	1 to 9,225
All Providers Avg	17.2 (27.8)	5.9	1 to 241.5
Primary Care	40.9 (46.7)	26.5	1 to 241.5
General Surgery	25.8 (33.8)	11.3	1 to 149.2
Cardiology	17.9 (38.7)	1.8	1 to 133.3
Pulmonary	15.1 (28.1)	1.2	1 to 104.0

Node Betweenness			
All Providers	0.005565 (0.042951)	0	0 to 0.658426
Primary Care	0.033479 (0.106154)	0.002952	0 to 0.658426
General Surgery	0.011113 (0.061974)	0	0 to 0.493710
Cardiology	0.002786 (0.009301)	0	0 to 0.040531
Pulmonary	0.001038 (0.003479)	0	0 to 0.013820

**Figure 3:**
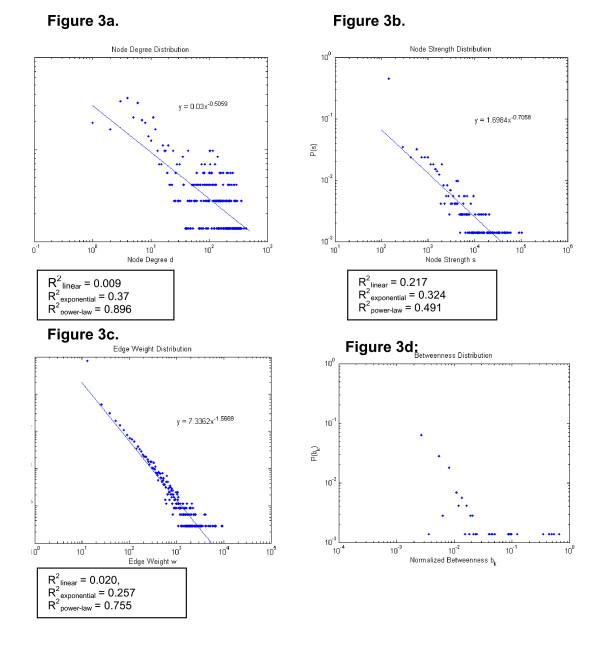
**Log-log plots of network metrics**.

To further investigate the overall properties of these networks, we constructed log-log plots of the distribution of each of the above network metrics. (Figure 3) The log-log plot of the edge weight distribution appears to be consistent with a scale-free network, while the distributions of node degree and node strength are less so, and are similar to a 'heavy-tail, droop-head' distribution, characteristic of networks that are formed by a combination of preferential and random attachments. The implications of these distributions for how the network forms, and how information flows across the network are found in the discussion below.

**Figure 4:**
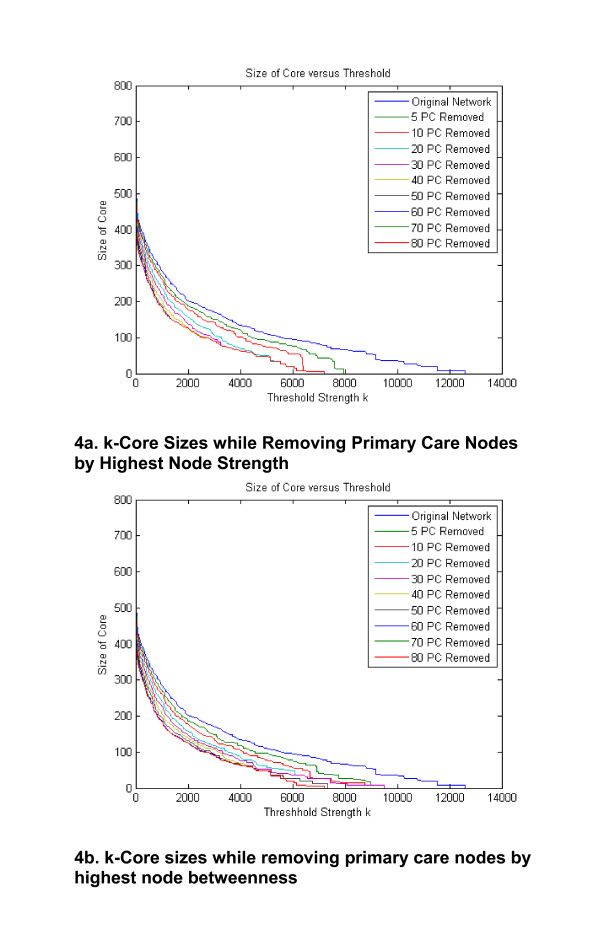
**The size of the network k-core versus the core threshold *k *by node strength as primary care nodes are removed from the network**.

Our analysis identified primary care type nodes hold important roles in connecting the network because of their relatively higher average node degree, node strength, and node betweenness compared to other provider node types. We observed how the sizes of the network cores change as all of the 80 primary care nodes in the network are removed one at a time from the network by rank order starting with the primary care node with the highest strength (Figure 4a). The top curve in Figure 4a corresponds to the original network with all primary care nodes, and the bottom curve corresponds to a second network with all primary care nodes removed. It can be seen that after 10 to 20 primary care nodes are removed, there is little change in the network cores as the remaining primary care nodes are removed. We repeated this analysis with removing the primary care node by order of the highest betweenness nodes first (Figure 4b). We observe that the results are mostly similar for the two removal strategies, but the removal by node strength reduces the core sizes more rapidly. This indicates that the node strengths are better than the node betweennesses for identifying critical central nodes for holding together the strongest cores of the network from among the primary care nodes.

## Discussion

We have demonstrated that the delivery of healthcare in a large healthcare system such as the VA can be represented as a complex network where provider nodes are linked by 'edges' formed by delivering care to the same patient, and that such a network has properties that reflect both preferential and random attachments. First, we discuss conceptual models for the spread or diffusion of a new physician behavior across a network. Next we discuss how some of the properties of the observed network are 'scale-free,' some are not, and implications of network structure for how information spreads across such a network.

One of the basic tenets of a network analysis comprised of individuals, a 'social network,' is that the structure of the network matters. That is, the outcomes of a node and its future behavior depend in part on its relative position within the network. There is a burgeoning field of such analyses in the organizational and social science literature as we attempt to better understand predictors of organizational performance and outcomes [11,20,21].

While there are many models and behavioral theories about changing behavior, one that has been widely used is Everett Rogers' diffusion of innovation theory, which postulates a series of steps for an individual: knowledge, persuasion, decision, trial, and adoption [22]. Although empirical research has demonstrated the importance of contacts through a social network in this process, there remain many unanswered questions about timing of adoption and the influence of the structure of the network on adoption in healthcare. Outside the field of healthcare delivery, much of the work on networks and diffusion was done by observing and measuring personal contacts and interactions. Over the past decade, much of that interpersonal communication and opinion leadership is mediated by two-way electronic media, such as an EMR [23].

Some may question the construction of a network based on the co-care of a patient as documented in an electronic health record (EHR). The question is whether merely observing behavior through a format such as the EHR will change physicians' behavior. The ability of people to influence each other without personal contact was recently demonstrated by Centola in an online social experiment [21]. Individuals were merely informed about the adoption of a health behavior by their online neighbors but were not allowed direct contact with them. The results showed that adoption of a new health behavior was much more likely when participants received social reinforcement as a result of belonging to a network characterized by many clustered ties but a high degree of separation, compared to those in a network where the connections between nodes are random. Thus, network structure has a profound effect on the dynamics of behavioral diffusion.

## Structural properties of the VA network

Unlike a binary network where a link is counted as only present or absent, an examination of the distributions of node strength and edge weight provides a better description of properties of a weighted network than node degree distribution. This is because the former properties reflect true nature of the network with weights on the links between nodes whereas node degree does not [13,14]. A close examination of resulting network plots in Figure 3, especially the edge weight distribution, suggests that this VA provider type/station network may have 'scale-free' characteristics. Scale-free networks have been observed in social, technical, and biologic networks [12,24,25]. For example, the number of sexual contacts follows a scale-free distribution within a society [24]. In a scale-free network the distribution of one or more metrics, in this case the edge weights and nearly the node strengths, follow a power-law distribution, thus the name scale-free. The existence of a power law means that edge weights have a wide distribution; there is no 'typical' or central tendency of the weight of the link between any two provider nodes. In a scale-free network, new information spreads rapidly across the network [23]. One example of this is the rapid spread of computer viruses through the internet, another scale-free network at the autonomous system level [25].

What are the implications of the scale-free distribution of edge weights within this network? If the propagation or implementation of new information or behaviors within a healthcare system were solely dependent on the strength of the link, the 'edge weight' between any two provider nodes, then a perfectly scale-free distribution of node strengths would suggest that the implementation of new evidence across a healthcare system would spread rapidly. Unfortunately, evidence suggests that is very rarely the case [26]. What other properties of the above described network might help us understand how new evidence is adopted in a healthcare system?

More recent work in network formation reveals that the 'heavy-tailed, droop-head' appearance in Figures 3a and 3b is a result of both preferential and random attachments governing the growth of the network [27]. The terms 'heavy-tail' and 'droop-head' refer respectively to the wide portion of the tail of the distribution (The distribution of node degrees from 20 to 429 diverge from the power-law model) and the lower values at the head of the distribution (The distribution between node degrees of 1 and 5 falls below the power-law model).

How might this occur within the delivery of healthcare? It is fairly obvious that within a healthcare system some constrained preferential attachments may be generated, for example, a primary care physician may be more likely to refer to a cardiologist that they know or with whom they have worked in the past within close geographic proximity. In addition, patients may be more likely to see another primary care provider they have heard about from other patients in the same clinic when their primary care provider is not available. The node strength serves as a measure of the popularity of the provider type node and as a significant influence in the development process of new connections among provider nodes.

But what about 'random' connections formed among provider nodes for some reason other than the wealth (node strength) of the other nodes in the network? There are several possibilities: some patients may move to another city and re-establish care at a different VA medical center resulting in a link between their former physician and new physicians at that distant VA medical center. It is also possible that patients seen by a primary care provider may become acutely ill and be seen by other physicians to whom the usual care physicians do not normally refer for this acute illness.

It has been shown in other network analyses that when the tail of the distribution is wide, there are physical limitations, such as geographic proximity of providers, that begin to influence the total number of contacts of a node [27]. The downward curve or 'droopiness' at the head of the distribution suggests that, in general, it is not desirable to be very poorly connected in the network, and thus the nodes at this end of the distribution form a few more connections to the rest of the network causing the frequencies of the least connected nodes to drop. Within a healthcare system such as the VA, providers are unlikely to be very poorly connected to the network because the patient population they care for are largely patients with multiple chronic medical conditions, requiring the service of a diverse group of specialists and primary care providers [28].

Because there are many factors (other than those that might be explained by a wealth of a provider-type at a clinic/station or the clinic/station itself) that may influence the decisions of providers in where they refer their patients, and thus whom they end up sharing patients with, some 'randomness' or deviations from a near-perfect power-law distribution is expected for the node degrees and strengths. However, from the nearness of Figure 3b to a power-law distribution (R^2 ^= 0.755), it appears that whatever the various factors are that influence the connections, most of them would correlate with the strengths of the provider nodes.

What are the implications of the node degree and node strength distributions for the diffusion of information across this network? The flow of information described above is slower in networks that are not highly scale-free [13,15]. This finding may partially explain why the spread or propagation of the use of new more effective medications or therapies or diagnostic tests occurs slowly across a healthcare system such as the VA.

As shown in Figure 4, the overall connectivity of the network would be much lower if only 10 to 20 specific primary care nodes were removed. There are two possibilities as a result of such a disruption. First, it is possible that such a disruption might affect the propagation of new information across the network. So although networks with scale-free properties are robust to random removal of nodes, targeted removal of 10 to 20 specific primary care nodes could severely restrain the ability of the network to spread new ideas or knowledge [29-31]. This finding also suggests that improving dissemination and implementation of evidence-based practice across the network might be accelerated by targeting changes in the behaviors of these major hubs on the network. Conversely, another type of information flow in a network is the sharing of 'normative values' which might be at odds with innovative practices. Edges convey normative values as well as new evidence-based ideas. Nodes with many connections might be a barrier to spread of a new behavior across the network if the normative values conflict with new evidence, especially if they are the ones who are the oldest and most resistant to change. Thus, removal of these nodes or reducing their connectiveness might actually enhance the adoption of new behaviors among clinicians.

Several limitations exist in our analysis. First, and perhaps most important, was the inability to obtain data such that nodes represented distinct individual providers rather than a type of provider within each clinic/station. It is possible that the centrality of primary care providers is an artifact of this limitation. Second, we were only able to construct a limited network in one small region of the US. A larger national data set would provide much greater insight into the network structure. Finally, we do not have outcome measures that would measure the spread or diffusion of evidence or change in behaviors across the network to test formal hypotheses regarding the influence of network properties on diffusion. Any such under-taking would also need to consider other network properties such as academic affiliation of VA medical centers.

## Conclusions

In conclusion, similar to other studies of complex systems, the delivery of healthcare in a large system such as the VA can be represented as components that interact to form a complex network. By using currently available tools to explore its topology, it should be possible to investigate how the underlying connectivity of such a system affects its behavior and develop strategies to improve its performance. For example, one might study diffusion of a provider behavior such as adoption of a new feature in the EHR by targeting initial implementation at key hubs identified in a network analysis. The Veterans Health Administrations continues to implement new features in their clinical information infrastructure such as new optional decision-making support or even use of secure email to communicate with patients. This would be a very rich and observable domain for a network analysis. In addition, the findings would leverage our understanding of how network properties may be used to improve quality and outcomes of care.

## Competing interests

The authors declare that they have no competing interests.

## Authors' contributions

PS carried out the network analysis and prepared the tables and figures for the manuscript. CS supervised the work on the network analysis, assisted with interpreting the network metrics and helped to draft the manuscript. MP conceived of the analysis, assisted with interpretation of the network data and drafted the manuscript. All authors read and approved the final manuscript.
